# Childhood tuberculosis outbreak in Africa: is it a matter of concern?

**DOI:** 10.1097/JS9.0000000000000140

**Published:** 2023-03-03

**Authors:** Olivier Uwishema, Anushree Rai, Aderinto Nicholas, Mortada Abbass, Lama Uweis, Sara Arab, Rayyan El Saleh, Irem Adanur, Daniel Stephen Masunga, Abubakar Nazir

**Affiliations:** a Oli Health Magazine Organization, Research and Education, Kigali, Rwanda; b Clinton Global Initiative University, New York, New York, USA; c Faculty of Medicine, Karadeniz Technical University, Trabzon, Turkey; d Chhattisgarh Institute of Medical Sciences, Bilaspur, Chhattisgarh, India; e Faculty of Medicine, Beirut Arab University, Beirut, Lebanon; f Department of Medicine and Surgery, Ladoke Akintola University of Technology, Ogbomoso, Nigeria; g Kilimanjaro Christian Medical University College (KCMUCo), Moshi, Tanzania; h Department of Medicine, King Edward Medical University, Lahore, Pakistan

Highlights

- Tuberculosis is caused by *Mycobacterium tuberculosis*. The bacterium is often transmitted through tiny droplets released into the air. The most common signs and symptoms are a severe cough lasting for more than 3 weeks, spitting of blood, and chest pain.
- Although it mainly affects adults, younger children suffering from malnutrition, HIV, and other immunosuppressive diseases may also be at a higher risk of contracting the infection.
- In endemic locations, efforts should be focused on obtaining a precise, accurate, and quick diagnosis of childhood tuberculosis. For better management in the pediatric age group and ultimately in the entire population, an improved diagnosis will result in the development of more accurate disease statistics and better epidemiological characterization.

## Introduction

Tuberculosis (TB) is a highly infectious yet curable and preventable disease that mostly affects the lungs^1^. An airborne disease, the TB bacterium is expelled when an infected individual sneezes or coughs, and one does not need more than inhaling the bacteria to get infected^1^. Usually, TB may stay dormant in patients’ bodies until years later, when their immunity weakens. However, some others develop TB within weeks of being infected, especially immunocompromised patients^2^. According to the Centers for Disease Control (CDC), the most common signs and symptoms of TB are a severe cough that lasts more than 3 weeks, spitting of blood, and chest pain^3^. TB is mainly a disease in adults, but people of any age may have it^1^. Thus children, especially those between 1 and 4 years of age^4^, are not protected. Dr Matshidiso Moeti, the WHO Regional Director for Africa, reports that ‘Any child living in a setting where there are people with infectious TB can become ill with TB, even if they are vaccinated’^4^.

The African region harbors around 17 out of 30 countries with the highest TB cases^5^. Moreover, according to the WHO regional office for Africa, approximately one million children were infected with TB, and 170,000 died because of TB in 2015^6^. The WHO End TB Strategy set a milestone which states that by 2020 TB cases should be reduced by 20% and deaths by 35%^5^. Unfortunately, only six countries managed to reach this milestone^5^. According to the latest numbers in 2022, Africa has around one-third (320,000 children) of all TB cases among children between 0 and 15 years of age worldwide^5^. Above all, it is assumed that about two-thirds of children are undiagnosed due to the lack of resources^5^. This means that the disease transmission to other children, and even adults, is accelerated by the undiagnosed patients, thus mortality rate is also increasing.

On 24 August 2022, the WHO along with the African Union (AU) raised the importance of finding quick and effective measures to put an end to the increasing number of TB cases among African children^5^.

## Epidemiology and outbreak of childhood TB in Africa

The African continent is home to 17 countries classified among the 30 countries with the highest prevalence of pediatric TB^5^. Affecting 322,000 cases of children and young adults, reaching about a third of TB cases in young people under 15 years of age worldwide, TB represents an important factor in morbidity and mortality of children in developing countries, such as most African countries^7^. Among the African countries, the Central African Republic, Namibia, Lesotho, and Gabon appear to be the highest incidence of TB in 2020, according to WHO statistics^8^. Recently, the number of TB cases among children is not a good reflection of the actual situation as most of the cases go undiagnosed or are not reported, and this leads to a major contribution to the spread of the disease and rapid progression of symptoms without proper medical intervention^5^. Furthermore, the course of the disease appears to be evoked by the prevalent malnutrition in most African countries, which leads to a severe form of TB after infection with *M. tuberculosis*, estimating that up to 19% of TB cases worldwide are associated with malnutrition^5^,^9^. The WHO estimates the burden of TB on the pediatric population taking annually the lives of 450,000 children in developing countries^9^. Moreover, the presence of TB-associated HIV-positive children aggravates the mortality risk, raising it to cause the death of every one in five children infected with HIV^10^. Highlighting this point, the epidemiologic distribution of TB among the pediatric population in Africa is the highest globally. Yet, multiple factors have contributed to the increase in the disease burden in recent years, including the novel coronavirus disease 2019 pandemic and the decrease in access to healthcare facilities, leaving the burden to face the growing outbreak on the healthcare system^11^.

**Figure:**
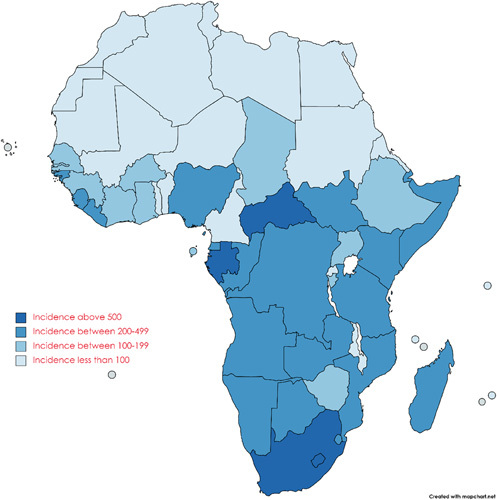


A map showing the incidence of TB (per 100,000 people) in Africa in the year 2020.

## Etiology of childhood TB

*M. tuberculosis* is the primary causative agent of TB. When an individual who has TB coughs, speaks or sings, the bacteria are released into the air and infect nearby individuals^5^. TB is most common in underprivileged and at-risk areas, directly resulting in further deprivation and heightened vulnerability for those populations^12^. Malnutrition is one of the exacerbating factors of TB. Worldwide, malnutrition is related to 19% of TB cases^13^. In many parts of the world, particularly those with high rates of HIV infection, TB continues to be a serious burden^14^. Reported HIV–TB co-infection rates to take into account current national HIV infection rates as well as other relevant variables like the degree of immune suppression. Similar to our study, several others on childhood TB report slightly more males than females as having the disease. According to reports, younger children are more likely to develop TB disease after contracting the infection^15^. The most common way that children contract TB is through infected adult contacts. Due to the high case density and extended diagnostic delay, high transmission rates are maintained in TB-endemic areas. Children are most severely impacted and the adult TB epidemic is not well controlled^16^. Particularly when their immune systems are weakened by malnutrition, HIV, or other diseases, children are much more likely to develop active TB, which increases the severity of the illness^17^. Younger ages, parents who were born abroad or in high-prevalence nations, HIV infection, and extended close contact with the index case are risk factors for infection in children. Smoking, being a young adult, having cavitary lesions, and having a positive sputum smear test are related index case risk factors. The time spent close to the TB patient is crucial. By taking into account maternal TB and sleep proximity, index case infectivity, duration of exposure, and exposure to multiple index cases, a contact-scoring tool estimated exposure quantification. In a setting with a high infection rate, it enabled the detection of 80% of the child’s infection risk^18^.

## Current efforts to mitigate childhood TB in Africa

In Africa, the prevalence of pediatric TB has risen recently. Currently, the region is responsible for 33% of all TB cases in children under 15 years worldwide^19^. This number might not be accurate because a large number of pediatric TB cases in Africa go unreported and untreated^18^. Due to staffing shortages and the challenge of accurately diagnosing children, the existing African health system finds it difficult to address the burden of childhood TB, even in instances that have been recorded^19^. There have been calls for an immediate reaction to the looming crisis to lessen its short-term and long-term effects^19^.

The AU and WHO issued a joint statement in August 2022 outlining their enhanced resolve to tackle the growing TB incidence in Africa. The call to action is an important step, particularly given the twin plagues of TB and undernutrition. It has been established that malnutrition makes children’s TB worse^13^. Malnutrition, sadly, is a widespread issue throughout Africa.

Additionally, there has been a rise in calls for more financing to fight TB. Malaria is currently the disease in Africa, receiving the most money^5^. Africa has very little financing for TB control, and without significant effort, the continent runs the risk of missing the 2030 eradication objective. Numerous stakeholders have argued for expanding the search for missed cases and incorporating pediatric TB into Africa’s present child health programs too to address this situation. The integration of TB into HIV services has seen some success. But incorporating this approach into more comprehensive child health services appears to be a key resolution^5^. A technique like this would enhance the creation of current recommendations and the accessibility of ready-to-use services and medications for children with TB.

## Recommendations

African nations need to provide more financial, technological, and human capital for TB prevention and control to hasten the disease’s eradication in children and adolescents^5^. The current level of funding and commitment in Africa’s fight against TB puts the world’s goal of eradicating the disease by 2030 at risk^5^. Africa needs at least US$1.3 billion annually for TB prevention and treatment, but only governments contribute 22% of that amount, with 34% coming from outside sources^5^. The remaining portion of the budget is underfunded^5^. The WHO also suggests that antibiotics susceptibility tests should be done as soon as possible, followed as closely as feasible by bacteriological confirmation^20^. Before treatment programs are developed, surveillance studies to assess the prevalence of TB have been mandated for every nation, region, and organization^21^. In addition, the most important component of controlling TB is secondary prevention^22^. Although it is aimed at each patient specifically, it is a crucial step in halting the transmission chain^20^. In endemic locations, efforts should be focused on obtaining a precise, accurate, and quick diagnosis of childhood TB^23^. For better management in the pediatric age group and ultimately in the entire population, an improved diagnosis will result in the development of more accurate disease statistics and better epidemiological characterization^23^. Childhood TB can be used to track the effectiveness of national TB control programs in the communities since childhood TB incidence is a predictor of adult contacts who have active TB^23^. In addition to developing standard operating procedures to accommodate pediatric specimens other than sputum, including gastric aspirates and cerebrospinal fluid, national recommendations must address the routine use of Xpert MTB/RIF on all children with suspected TB^23^. Children under the age of 5 should be included as test subjects in clinical trials for the development of new medications and vaccines^23^.

## Conclusion

TB is caused by *M. tuberculosis* which is transmitted through tiny droplets released into the air via cough. The most common signs and symptoms of TB are a severe cough that lasts more than 3 weeks, spitting of blood and chest pain. Although it mainly affects adults, younger children suffering from malnutrition, HIV and other immunosuppressive diseases may also be at a higher risk of contracting the infection. Furthermore, due to high case density and extended diagnostic delay, high transmission rates are still maintained in TB-endemic areas.

TB represents an important factor in the morbidity and mortality of children in African countries and is home to 17 countries classified among the 30 countries with the highest prevalence of pediatric TB. Although the AU and WHO have issued a joint statement outlining their resolve to tackle the growing TB incidence, African nations still need to provide more financial, technological, and human capital to hasten the disease’s eradication. Several recommendations such as mandatory surveillance, antibiotic susceptibility testing, secondary prevention, development of efficient vaccines, and precise and quick diagnosis have been mentioned for more accurate disease statistics and better epidemiological characterization of childhood TB.

**Figure:**
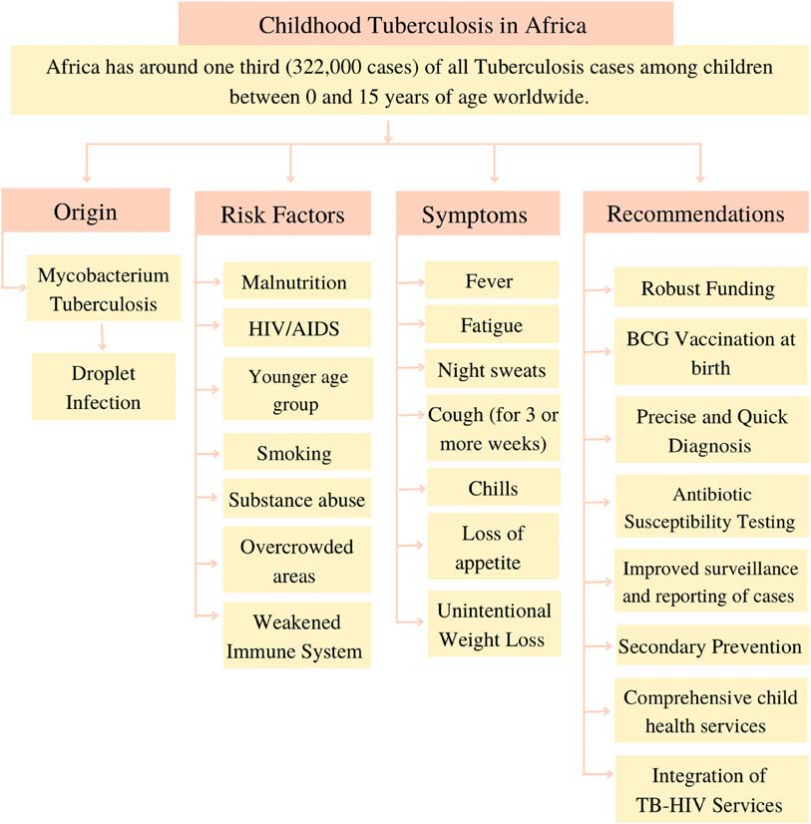


This figure was drawn and analyzed by authors Anushree Rai and Olivier Uwishema.
